# The influence of orthopedic corsets on the incidence of pathological fractures in patients with spinal bone metastases after radiotherapy

**DOI:** 10.1186/s12885-015-1797-5

**Published:** 2015-10-20

**Authors:** Harald Rief, Robert Förster, Stefan Rieken, Thomas Bruckner, Ingmar Schlampp, Tilman Bostel, Jürgen Debus

**Affiliations:** 1 Department of Radiation Oncology, University Hospital of Heidelberg, Im Neuenheimer Feld 400, 69120 Heidelberg, Germany; 2 Department of Medical Biometry, University Hospital of Heidelberg, Im Neuenheimer Feld 305, 69120 Heidelberg, Germany

## Abstract

**Background:**

Clinical care of unstable spinal bone metastases in many centers often includes patient immobilization by means of an orthopedic corset in order to prevent pathological fractures. The aim of this retrospective analysis was to evaluate the incidence of pathological fractures after radiotherapy (RT) in patients with and without orthopedic corsets and to assess prognostic factors for pathological fractures in patients with spinal bone metastases.

**Methods:**

The incidence of pathological fractures in 915 patients with 2.195 osteolytic metastases in the thoracic and lumbar spine was evaluated retrospectively on the basis of computed tomography (CT) scans between January 2000 and January 2012 depending on prescription and wearing of patient—customized orthopedic corsets.

**Results:**

In the corset group, 6.8 and 8.0 % in no-corset group showed pathological fractures prior to RT, no significant difference between groups was detected (*p* = 0.473). After 6 months, patients in the corset group showed pathological fractures in 8.6 % and in no-corset group in 9.3 % (*p* = 0.709). The univariate and bivariate analyses demonstrated no significant prognostic factor for incidence of pathological fractures in both groups.

**Conclusions:**

In this analysis, we could show for the first time in more than 900 patients, that abandoning a general corset supply in patients with spinal metastases does not significantly cause increased rates of pathological fractures. Importantly, the incidence of pathological fracture after RT was small.

## Background

Spinal bone metastases represent the most frequent site of skeletal metastases [1]. The effects of bone metastases are a major concern in everyday clinical practice and result in pain at rest and during activity, limitations in daily life, lower performance ability, risk of pathological fractures and neurologic deficits [2], with a significant reduction in the patients’ quality of life (QoL). Radiotherapy (RT) is the most common treatment option of bone metastases in advanced tumor disease [3]. The aim of therapy hereby is to reduce pain, to improve the functionality, and to prevent complications, for example compression of the spinal cord and pathological fractures. Pathologic fractures occurred in 39 % of patients with breast cancer, in 22 % of patients with prostate cancer, and in 22 % of patients with bone metastases from lung cancer or other solid tumors during 12, 15, and 21 months of follow up, respectively [4, 5]. Consequently, pathologic fractures are a significant clinical concern in these patient populations, and preventing or delaying fractures is an important treatment objective. In previous retrospective studies among American and Japanese populations, the incidence of pathologic fractures in the vertebral column is estimated to range at 10 % [6, 7]. Clinical care of unstable metastases in many centers often includes patient immobilization either by means of an orthopedic thoracic corset or by confining the patient to bed in order to prevent pathological fractures, which further decreases patients` QoL. Accordingly the incidence of pathological fractures after RT in patients with spinal bone metastases while wearing an orthopedic corset is still unknown. The aim of this retrospective analysis was to evaluate the incidence of pathological fractures after RT in patients with and without orthopedic corsets and to assess prognostic factors for incidence of pathological fractures in patients with spinal bone metastases.

## Methods

A cohort of 915 patients, was treated by RT for osteolytic metastases of the vertebral column due to histologically diagnosed solid tumors at the University Clinic of Heidelberg in the period from January 2000 until January 2012. All patients were examined using computed tomography scans (CT) in this retrospective analysis. Inclusion criteria were an osteolytic phenotype, location in the thoracic or lumbar spine and a minimum duration of follow-up treatment of 6 months. A total of 2.195 bone lesions in the thoracic and lumbar spine were identified. Bone metastases diagnoses were verified by CT. The patient data were taken from the Heidelberg NCT Cancer Registry and are summarized in Table 1. Performance status was expressed using the Karnofsky Performance Score (KPS) [8]. The specifications for an unstable vertebral body were tumor occupancy of more than 60 % of the vertebral body, and pedicle destruction [9]. Patients with an orthopedic corset used a thoraco-lumbo-sacral orthosis (TLSO) brace. The prescribed corset was prophylactically with no relation to existence of a pathological fracture. The pathological fractures were evaluated in the irradiated spinal region. New diagnosed fractures were analyzed prior to RT and 6 months after RT. This study was approved by the Heidelberg Ethics Committee on 22 October 2012 (nr S- 513/2012).

**Table 1:** Patient characteristics

		Corset group		No corset group		All	
		*n*	%	*n*	%	*n*	%
Age (mean, SD)		63.2 (+/− 11.4)		62.2 (+/−10.8)		62.7 (+/−11.1)	
Gender	male	236	53.4	253	53.5	489	53.4
	female	206	46.6	220	46.5	426	46.6
KPS	<=70	204	46.2	218	46.1	422	46.1
	>70	238	53.8	255	53.9	493	53.9
Primary site	NSCLC	101	22.9	206	43.5	425	46.5
	Breast	219	49.6	74	15.6	175	19.1
	Kidney	71	16.1	88	18.6	159	17.4
	Melanoma	15	3.4	26	5.5	41	4.5
	Prostate	4	0.9	13	2.8	17	1.9
	Other	51	11.5	105	22.2	98	10.6
Localization	Thoracic	284	64.3	279	59.0	563	61.5
	Lumbar	158	35.7	194	41.0	352	38.5
Chemotherapy	yes	242	54.7	260	55.1	502	54.9
	no	200	45.3	212	44.9	412	45.1
Stability before RT	stable	140	31.7	320	67.7	460	50.3
	unstable	302	68.3	153	32.3	455	49.7
Stability after 3 months	stable	152	39.8	279	79.0	449	59.2
	unstable	230	60.2	79	21.0	309	40.8
Stability after 6 months	stable	165	45.1	311	85.2	476	65.1
	unstable	201	54.9	54	14.8	255	34.9
Bisphosphonates	yes	351	79.4	296	62.6	647	70.7
	no	91	20.6	177	37.4	268	29.3
Distant metastases	Brain	50	11.3	77	16.3	127	13.9
	Lung	74	16.7	118	25.0	192	21.0
	Liver	70	15.8	116	24.5	186	20.3
	Skin	11	2.5	25	5.3	36	3.9
Number of metastases	solitary	189	42.8	228	48.2	417	45.6
	multiple	253	57.2	245	51.8	498	54.4

*SD* Standard deviation; *KPS* Karnofsky performance score; *RT* Radiotherapy

### Statistical analysis

The empirical distribution of continuous variables is described by the number of observations, mean and standard deviation; the description of categorical variables includes the number and percentage of patients belonging to the relevant categories. We estimated number of observations of pathological fractures before and 6 months after RT and compared them between groups according to the chi-square test. The univariate log-rank test was used to evaluate the prognostic importance for occurrence of pathological fractures of gender, Karnofsky performance score, non-small cell lung cancer (NSCLC), breast cancer, kidney cancer, localization of metastases, chemotherapy prior to RT, stability prior to RT, stability after 3 months, stability after 6 months, bisphosphonates, and number of bone metastases. Results were reported as *p*-values of the logrank tests. Bivariate analysis was performed to detect factors independently associated with pathological fractures using a Cox regression model. This regression analysis was performed including gender (male), Karnofsky performance score (<=70), NSCLC (no primary), breast cancer (no primary), kidney cancer (no primary), localization of metastases (thoracic), chemotherapy prior to RT (no chemotherapy), stability prior to RT (unstable), stability after 3 months (unstable), stability after 6 months (unstable), bisphosphonates (no bisphosphonates), and number of bone metastases (solitary metastasis). The results are reported as *p*-values, odds ratios and 95 % confidence intervals (CI). For all analyses, a *p*-value of 0.05 or less was considered significant. All statistical analyses were done using the SAS software version 9.3 (SAS Institute, Cary, NC, USA).

### Radiotherapy

RT was performed in the Department of Radiation Oncology at the Heidelberg University Clinic. After virtual simulation was performed to plan the radiation schedule, RT was carried out over a dorsal photon field of the 6MV energy range. The photon field covered the specific vertebral body affected as well as the ones immediately above and below. The median individual dose in all patients was 3 Gy (range 2–3 Gy), the median total dose 30 Gy (range 20–35 Gy). The individual and total doses were decided separately for each individual patient, depending on histology, the patient’s general state of health, the current staging, and the corresponding prognosis.

## Results

The mean follow-up was 6.3 months for both groups. Of all patients, 31.7 % (140 patients) in the corset group and 67.7 % (320 patients) in the no-corset group were classified as stable prior to RT, 79.4 % (*n* = 351) and 62.6 % (*n* = 296) of the corset and no corset group were also treated with bisphosphonates. Considering the number of metastases, 57.2 % (*n* = 253) in the corset group and 51.8 % (*n* = 245) in the no corset group showed multiple metastases. The incidence of unstable metastases was higher in the corseted group (68.3 %) compared to the non-corseted group (32.3 %) prior to RT (*p* < 0.01). The incidence of pathological fractures prior to RT was 7.4 % in all patients. In the corset group, 6.8 and 8.0 % in no-corset group showed pathological fractures prior to RT, no significant difference between groups was detected (*p* = 0.473). After 6 months, the fracture rate was in total 9.0 % for all patients and correspond 1.6 % new diagnosed fractures. Patients in corset group showed in 8.6 % and in no-corset group in 9.3 % pathological fractures (correspond 1.8 and 1.3 % new diagnosed fractures) (*p* = 0.709). The thoracic spine showed more fractures significantly (Table 2).

**Table 2:** Pathological fractures before and after RT

		All		Corset group		No corset group		*p*-value
		*n*	%	*n*	%	*n*	%	between groups
Pathological fracture	yes	68	7.4	30	6.8	38	8.0	
before RT	no	847	92.6	412	93.2	435	92.0	0.473
	thoracic	42	61.8	22	73.3	20	52.6	
	lumbar	26	38.2	8	26.7	18	47.4	0.081
Pathological fracture	yes	82	9.0	38	8.6	44	9.3	
after 6 months	no	833	91.0	404	91.4	429	90.7	0.709
	thoracic	51	62.2	28	73.7	23	52.3	
	lumbar	31	37.8	10	26.3	21	47.7	0.046

Pathological fractures before and 6 months after RT in corset and no corset group

The univariate and bivariate analyses identified no significant prognostic factors for incidence of pathological fractures in both groups (Tables 3 and 4).

**Table 3:** Univariate analysis of prognostic factors for incidence of pathological fractures

Factor	Corset group	No corset group
	*p*-value	*p*-value
Gender	0.357	0.642
KPS	0.402	0.819
NSCLC	0.101	0.184
Breast cancer	0.496	0.091
Kidney cancer	0.072	0.195
Localization	0.205	0.342
Chemotherapy	0.454	0.379
Stability before RT	0.268	0.677
Stability after 3 months	0.513	0.614
Stability after 6 months	0.202	0.402
Bisphosphonates	0.183	0.420
Number of metastases	0.145	0.229

**Table 4:** Bivariate analysis of prognostic factors for incidence of pathological fractures

	All			Corset group			No corset group		
	OR	95 % CI	*p*-value	OR	95 % CI	*p*-value	OR	95 % CI	*p*-value
Gender	0.797	0.503–1.262	0.333	0.727	0.369–1.434	0.358	0.862	0.461–1.612	0.642
KPS	0.843	0.535–1.327	0.461	0.753	0.387–1.465	0.403	0.930	0.500–1.731	0.819
NSCLC	0.604	0.376–0.969	0.036	0.566	0.285–1.126	0.105	0.645	0.336–1.237	0.186
Breast cancer	0.562	0.284–1.113	0.098	0.745	0.318–1.746	0.498	0.369	0.111–1.225	0.103
Kidney cancer	1.071	0.595–1.928	0.818	2.008	0.928–4.345	0.076	0.534	0.204–1.398	0.202
Localization	0.970	0.607–1.548	0.897	0.618	0.292–1.308	0.208	1.351	0.725–2.517	0.343
Chemotherapy	1.020	0.647–1.609	0.931	1.295	0.657–2.555	0.455	1.329	0.704–2.511	0.381
Stability before RT	0.937	0.595–1.474	0.777	0.647	0.298–1.406	0.271	1.155	0.586–2.276	0.677
Stability after 3 months	0.993	0.594–1.660	0.980	0.776	0.363–1.660	0.514	1.266	0.505–3.174	0.614
Stability after 6 months	0.939	0.537–1.642	0.826	0.587	0.256–1.343	0.207	1.682	0.493–5.740	0.406
Bisphosphonates	0.939	0.573–1.538	0.803	0.606	0.288–1.274	0.186	1.313	0.676–2.550	0.421
Number of metastases	1.314	0.826–2.090	0.249	1.689	0.829–3.442	0.149	0.683	0.365–1.277	0.232

## Discussion

Bone metastases are common in patients with advanced malignancies. The spinal column is the most common site of bone metastases. Metastatic vertebral body collapse is one of the major causes of severe back pain and neurologic compromise. Therefore, prevention of pathologic fractures is of clinical importance to maintain patients` QoL.

Bone metastases from solid tumors can dramatically increase bone resorption, resulting in skeletal complications such as pathologic fractures (10–20 % of patients), spinal cord compression (5 % of patients), hypercalcemia of malignancy (10–15 % of patients), severe bone pain requiring palliative RT, and represent important clinical issues. Fractures may cause severe bone pain, limit mobility, and require surgery and hospitalization for treatment [10, 11].

Our results showed a pathological fracture rate of 7.4 % in all patients. First, we compared between orthopedic corset and no-corset groups to examine the effectiveness of the corset for prevention of a pathological fracture. The corset group with 6.8 and 8.0 % in the no-corset group did not differ between groups. Additionally, after 6 months no significant difference between groups was detected. In a recent trial, the results showed a pathological fracture rate in 18 % of the vertebral bodies prior to RT. New fractures up to 6 months after therapy were seen in 2 % of all cases [12]. This 6-months fracture rate was comparable to our results. In previous retrospective studies among American and Japanese populations, the incidence of pathologic fractures in the vertebral column ranges around 10 % [6, 7] and corresponds to our findings. In a further analysis by Saad et al. [13], the risk of pathological fracture in association with lung cancer is given at 17 %; this finding, however, was made relative to the entire skeletal system. Pathological fractures are a frequently encountered event; fractures of the vertebral body following RT, on the other hand, are rarely reported. In our results, the thoracic spine showed significant more fractures 6 months after RT. However, 61.5 % of metastases were detected in the thoracic spine. The rib cage and sternum can provide additional structural support, but we could not detect any influence to our results. In the clinical treatment of spinal metastases, many advances have been made in the ability to determine both the size and location of vertebral lesions [14, 15]. Elevated risk of pathologic vertebral body fracture may not, by itself, justify prophylactic stabilization, as many fractured vertebrae are stable or fractured in a manner that does not compromise the spinal canal [2]. Increased tumor size, lower BMD, increased load, and pedicle involvement elevate the risk of burst fracture prior to endplate failure [15]. The study by Taneichi et al. [9] defines the risk factors for fractures of the vertebral bodies caused by osteolytic metastases and rates the estimated fractures according to different types of metastatic involvement, establishing criteria for assessing the risk of vertebral-body fractures. The risk factors for vertebral-body fractures in the thoracic region (T1-T10) are the tumor size and the degree of destruction of the costovertebral joint; in the thoracolumbar and lumbar region (T10-L5), it is the tumor size and degree of pedicle destruction that are the main factors [16]. Therefore, Taneichi et al. [9] conducted radiographic analyses of patients with metastatic spinal tumors and concluded that destruction of the costovertebral joint was one of the major risk factors of vertebral collapse in the thoracic and lumbar spine. Certainly, there are multiple scoring systems to assess spinal instability in the literature [17, 18]. We used the Taneichi Score because of the practicability and easy appliance in the clinical practice. As a main concern, this scoring system constitutes a simple method for classifying osteolytic metastases in vertebral bodies as „stable“or „unstable“by definition of risk factors such as tumor size and the degree of costovertebral joint destruction for the thoracic region (Th 1 to 10) and tumor size and the degree of pedicle destruction for the lumbar region (Th 11 to L5), which is why this score is employed in this evaluation. Reductions in fracture risk following bisphosphonate treatment are also frequently disproportionate to changes in bone density [19, 20]. Bisphosphonates were found to reduce the overall risk of skeletal complications by 14 % and to reduce the incidence of fractures by 28 to 37 % [21]. According to our data, both groups had a high bisphosphonates rate so that this bias was negligible small. Recently, however, zoledronic acid has demonstrated efficacy in the management of bone pain and prevention of SREs, including pathologic fractures [19]. However, bisphosphonate therapy was not a prognostic factor for pathological fractures according to our results.

Pathological fractures play a major role in everyday clinical practice. Common clinical care of unstable metastases or existing fractures often includes patient immobilization either by means of an orthopedic corset or by confining the patient to bed in order to prevent pathological fractures, which further decreases patients` quality-of-life (QoL). Secondly, the pain, which can be severe, is mechanical in origin, and frequently the patient is only comfortable when lying still. However, no data regarding the appearance of pathological fractures by wearing a corset exist so far. According to our results, patients without an orthopedic corset did not have an increased risk of pathological fractures after RT. In both groups unstable metastases were detected, while the number in the corset group was significantly higher. In our opinion, wearing a corset also maintains some disadvantages in palliative patients: atrophy of the paravertebral musculature, limitation in mobility and reduction of the QoL. The importance of the corset in stability-endangering metastases was discussed up to now controversially, whether thereby fractures can be avoided. The vertical pressure load on the vertebral body also continues with corset, only axial movements can be decreased. Some prognostic factors as mentioned above are already known, our analysis could not identify prognostic factors for both groups. Only no NSCLC as primary site in all patients was significant, however, this result was affected due to a large number of evaluated NSCLC patients. Therefore, this point cannot be derived a factor. The main limitation of this study was the higher incidence of unstable vertebral bodies in patients in the corseted group (68.3 %) compared to the non-corseted group (32.3 %). Further limitations were the variety of primary tumors and the exclusion of patients presenting with cervical spine metastases. Among the strengths of our analysis were the large cohort and the very pertinent clinical question for patients with spinal bone metastases. This was, to our knowledge, the very first analysis to determine if a corset had any utility in preventing the incidence of pathologic fractures after routine radiotherapy.

## Conclusion

In this analysis, we could show for the first time in more than 900 patients, that by omission of corsets the fracture rate was not increased. Importantly, the incidence of a pathological fracture after RT was seldom. Large randomized trials are necessary to confirm these findings.
